# Insight into the cryptic diversity and phylogeography of the peculiar fried egg jellyfish *Phacellophora* (Cnidaria, Scyphozoa, Ulmaridae)

**DOI:** 10.7717/peerj.13125

**Published:** 2022-03-31

**Authors:** Carlos J. Moura, Nikolai Ropa, Bruno Ivo Magalhães, João M. Gonçalves

**Affiliations:** Okeanos-UAc–Research Institute in Marine Sciences, University of the Azores, Horta, Azores, Portugal

**Keywords:** Phylogeography, Systematics, DNA barcoding, Macaronesia, Azores, Semaestomeae, Scyphozoa, Jellyfish blooms, Marine biogeography, Cryptic species

## Abstract

The fried egg jellyfish *Phacellophora camtschatica* (*senso lato*) is a morphologically peculiar and conspicuous species occurring mostly in the cold waters of the North Pacific. It is less common in the cold waters of the NW Atlantic, and occasionally has been reported in the Mediterranean, Arctic, East and South Pacific, and E, SW and NE Atlantic. However, sightings of this scyphozoan jellyfish have intensified during the past two to three decades in Macaronesia, the Iberian Peninsula and the Mediterranean. These jellyfish are known to be voracious predators of other jellies, but also of other taxa, including fish of commercial interest. Therefore, *Phacellophora* aggregations may threaten local fisheries, aquaculture, and local biodiversity structuring. We report the first known occurrences of *Phacellophora* in the Azores Islands, which apparently become more frequent in recent years of the past decade. We confirm, through DNA barcoding of COI and 16S mitochondrial markers, the genetic identity of *Phacellophora* occurring in the Azores (NE Atlantic). We reveal, with COI sequence data, three (potentially four) cryptic species within the *Phacellophora camtschatica* complex. Two *Phacellophora* species co-occur in the North Pacific. In the North Atlantic (and possibly in the Mediterranean) one or two distinct species exist. Three nominal species of the genus that are currently synonymized, with type localities in the N Pacific, NW Atlantic, and the Mediterranean, need reassessment. The morphotypes previously defined for the four putative species names given for *Phacellophora* might be eventually differentiated by the number and disposition of the marginal lappets of umbrellae. This morphologic character has to be further inspected in vouchers of the four genetic lineages of *Phacellophora*, to decide between the description of new species, and the resurrection of junior synonyms through the designation of neotypes with DNA Barcodes, to validate the identity of the cryptic taxa detected. More haplotype sampling is necessary across the distribution of the genus to further investigate the genetic diversity and phylogeographic history of *Phacellophora*. The high genetic relatedness of *Phacellophora* from the cold NW Atlantic and the sub-tropical shores of the Azores, revealed by 16S and COI sequence data, suggests a recent invasion, in terms of geologic time, of the temperate waters of the NE Atlantic (and possibly of the Mediterranean). The medusivorous habits of *Phacellophora*, and especially its predation on the mauve stinger (*Pelagia* spp.) which frequently blooms in Macaronesia and Mediterranean waters, could relate to the recent reports of *Phacellophora* in the Azores, Madeira, Canary Islands, and the Mediterranean.

More investment, including on scientific staff, is necessary to catalog, DNA barcode and monitor jellyfish dynamics more accurately worldwide.

## Introduction

“True jellyfish” are cnidarians of the class Scyphozoa that presently represent 235 nominal species recognized (WoRMS Editorial Board, 2021). These animals are key organisms in marine ecosystems. They interact in trophic chains either as voracious predators (*e.g.*, of planktonic organisms, crustaceans, small fishes, and of fish eggs and larvae), or as prey of many taxa including important emblematic and commercial species (*e.g.*, turtles, tuna, swordfish, and seabirds) (*e.g.*, Pauly et al., 2009; Doyle et al., 2014). They also represent an important food source to scavengers (*e.g.*, Sweetman et al., 2014).

Mass aggregations of jellyfish may occur naturally (*e.g.*, Benovic & Lucic, 2001; Hamner & Dawson, 2009), and although more long-term monitoring is needed (Condon et al., 2013), there is a general perception that proliferations may be increasing in frequency worldwide (Richardson et al., 2009; Brotz et al., 2012; Condon et al., 2013), impacting human activities such as fisheries (*e.g.*, Dong, 2018; Dong, Liu & Keesing, 2010), aquaculture (*e.g.*, Bosch-Belmar et al., 2021) and leisure/tourism (*e.g.*, Fautin, 2009). Jellyfish proliferations have been correlated to anthropogenic actions such as overfishing, climate change, eutrophication, habitat destruction/modification, and inadvertent introductions of exotic species (Mills, 2001; Purcell, Uye & Lo, 2007; Richardson et al., 2009; Lynam et al., 2011). Despite the profound impacts of jellyfishes in ecosystems, their morphological identification can be challenging because of their complex and fragile forms, and because of their life cycles that involve both pelagic (planula larva, ephyra, and medusa) and benthic phases (scyphistoma and strobila) (Strand & Hamner, 1988). The relatively recent use of molecular techniques is providing insights on systematic relations, overcoming the “taxonomic impediments” to study jellyfish. In particular, DNA barcoding, which employs a standard molecular marker (usually COI, but alternatively also 16S for Medusozoa) for species identification, has unveiled much cryptic diversity (*e.g.*, Hebert et al., 2003; Hebert & Gregory, 2005; Moura et al., 2008; Moura et al., 2018) including in scyphozoans (*e.g.*, Dawson & Jacobs, 2001; Gómez-Daglio & Dawson, 2017; Lawley et al., 2021).

Semaeostomeae scyphozoans represent the most common and well-known jellyfishes. While this order is not the most species-rich of scyphozoans, currently with 75 nominal species recognized (WoRMS Editorial Board, 2021), many of these are presumably widely distributed, but there is growing evidence of extensive cryptic diversity, and that most species are restricted to a region or ocean basin (*e.g.*, Dawson & Jacobs, 2001; Schroth et al., 2002; Lawley et al., 2021).

**Figure 1:**
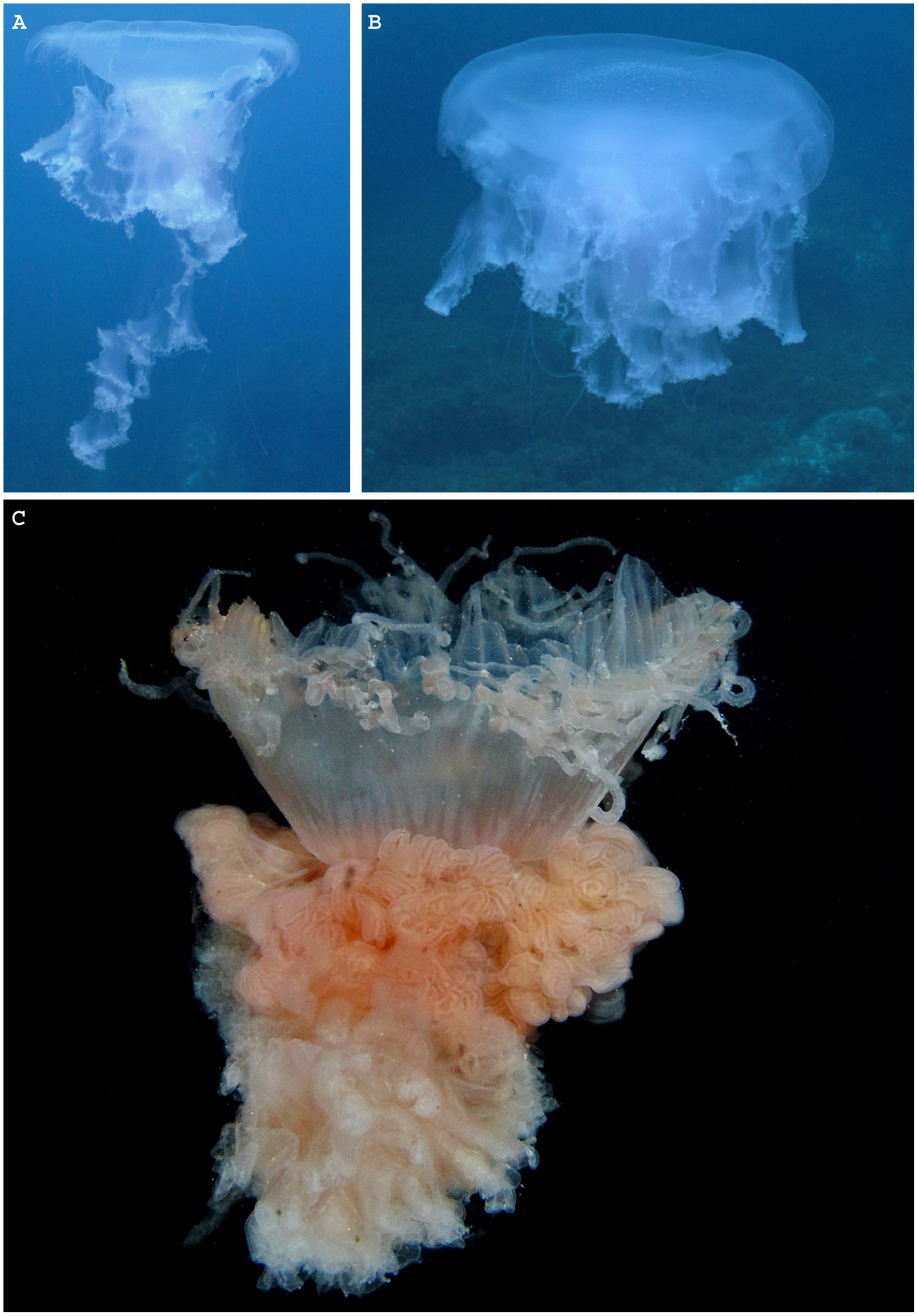
*Phacellophora* fried egg jellyfish in the Azores. (A, B) Specimen collected 19^th^ June 2020, (C) First *Phacellophora* reported in the Azores, in 2012 (note: identification not checked with laboratory analyses). Photos credits: Bruno I. Magalhães (A, B) & Nelson Raposo (C).

**Figure 2:**
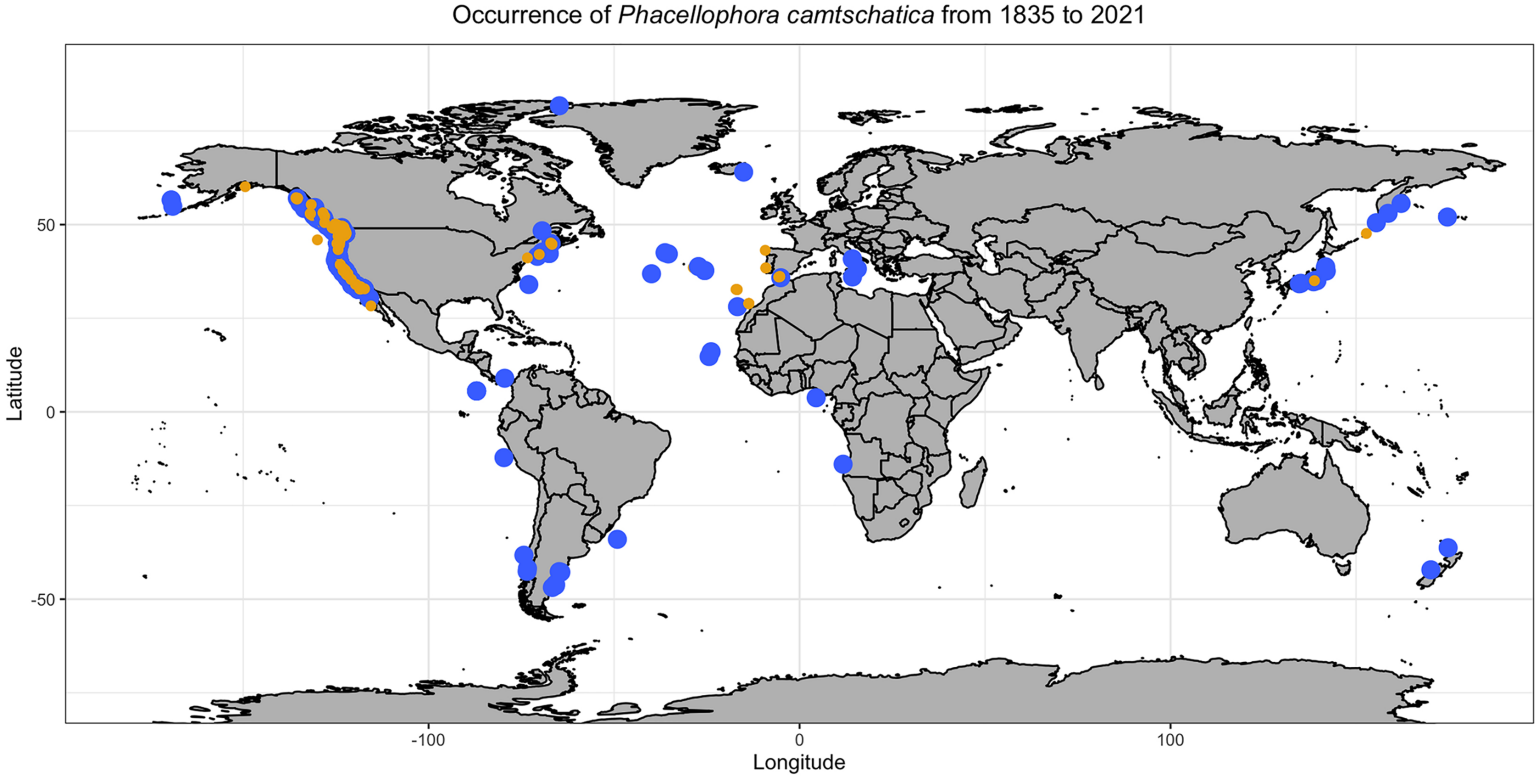
Map representing observations of *Phacellophora* worldwide. Blue points include scientific reports and museum samples. Yellow points represent *Phacellophora* reports in social networks and citizen science initiatives. Details of observation points are in Table S1.

**Figure 3:**
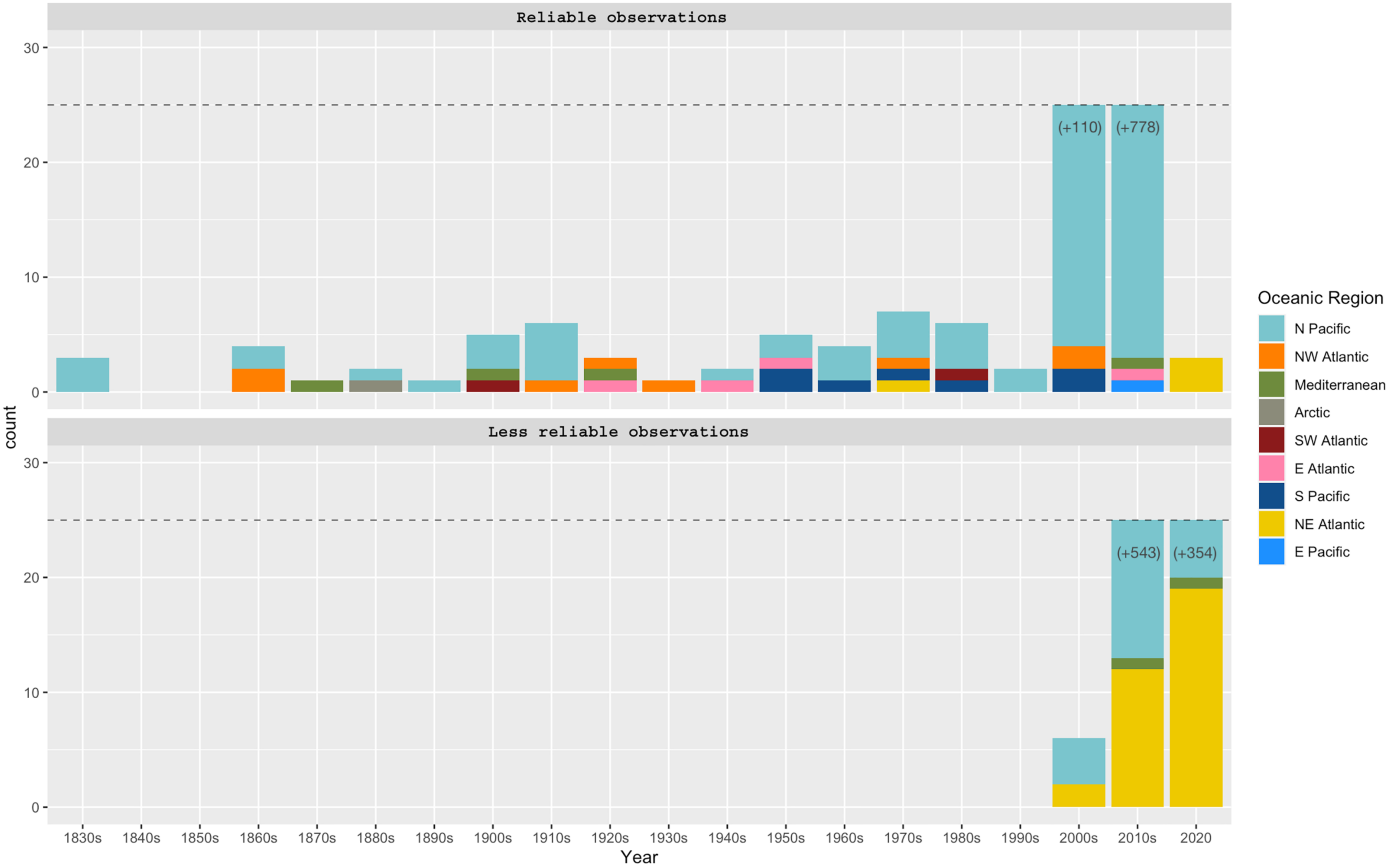
*Phacellophora* observations per decade and oceanic region. “Reliable observations” include scientific reports and museum samples. “Less reliable observations” refer to reports of social networks and citizen science initiatives. Details of observation points are in Table S1.

The Semaeostomeae is presently subdivided into five families (WoRMS Editorial Board, 2021), including the monotypic family Phacellophoridae that Straehler-Pohl, Widmer & Morandini (2011) designated to accommodate the peculiar “fried egg” jellyfish *Phacellophora camtschatica* (Brandt, 1835) (Fig. 1). However, this recent removal of the genus *Phacellophora* from the family Ulmaridae to the monogeneric family Phacellophoridae, based solely on morphologic characters of juvenile stages, was questioned by Gómez-Daglio & Dawson (2017), who argued, based on molecular data, that *Phacellophora* and the deep-water genus *Poralia* should instead belong to the subfamily Sthenoniinae of Ulmaridae, aside to the subfamilies Aureliinae and Deepstariinae, as accepted before Larson (1986). While *Phacellophora camtschatica* is currently the only species recognized in its family, it has three junior synonyms (after Bigelow, 1913 and Fedele, 1937a; Fedele, 1937b; Fedele, 1938), namely: *Phacellophora ornata* (Verrill, 1869), *Phacellophora ambigua* (Brandt, 1838), and *Phacellophora sicula* (Haeckel, 1880). Before this synonymy was accepted, *Phacellophora camtschatica* (type locality in the Kamchatka Peninsula, Russia) was thought to be distributed across the North Pacific between Siberia and California, *P. sicula* (type locality near Messina, Italy) was thought to be present in the Mediterranean and off the coast of Japan, *P. ambigua* (type locality off Washington) was thought to be distributed along the Pacific coast of North America, and *P. ornata* (type locality off Maine) was considered to be distributed in the cold waters of the NW Atlantic and SW Atlantic (Mayer, 1910). Later, *Phacellophora* continued to be observed in great numbers in the chilly waters of the N Pacific and NW Atlantic (*e.g.*, Brodeur et al., 2006; Zavolokin, Glebov & Kosenok, 2008; Il’inskii & Zavolokin, 2011), and much more sporadically in the SE, SW and E Pacific (Fagetti, 1973; Larson, 1986; Corrales-Ugalde et al., 2017), W Africa (Stiasny, 1934, 1940; Kramp, 1955, 1961; Hoving et al., 2018), Mediterranean (Dragičević et al., 2019) and NE Atlantic (*e.g.*, Stiasny, 1940; Moro et al., 2020; this study) (Figs. 2, 3).

Observations of *Phacellophora* in temperate/subtropical waters of the NE Atlantic during the past two to three decades, especially recent sightings in areas where this taxon had not been observed before, such as the Canary Islands (Moro et al., 2020), Madeira (Wirtz, 2021), the Iberian Peninsula (observations compiled here) and the Azores (this study; Fig. 3) is intriguing.

*Phacellophora camtschatica* s.l. (**sensu* lato*), commonly known as the “fried egg” (or “egg-yolk”) jellyfish, has a peculiar appearance, resembling a freshly cracked egg, and may attain 60 cm of bell diameter (see morphologic features in Mayer, 1910). It is more often encountered near the surface, exhibiting weak swimming capabilities, suggesting its distribution and aggregations are mainly driven by oceanic currents (Suchman & Brodeur, 2005). However, *Phacellophora* may exhibit active swimming behaviors in diel vertical migrations (Moriarty et al., 2012) reaching at least a depth of 750 m (Decker et al., 2014), which could partially explain its near cosmopolitan distribution (Fig. 2). On another hand, *Phacellophora* polyps have not yet been observed in natural habitats (Schiariti et al., 2018) and their habitat preferences could not impact distributions. The observation of small medusae in mesopelagic layers implies that the polyp phase of this taxon develops naturally in deep waters (Uye & Brodeur, 2017). If true, this would suggest that it is unlikely that *Phacellophora* has dispersed on ship-hulls or other floating materials during the polyp phase.

Due to its active feeding behavior, large size, wide curtain-like oral arms, and 16 clusters of long tentacles, *Phacellophora* is considered a voracious predator (Hamner & Dawson, 2009) with the potential to regulate planktonic communities locally (Strand & Hamner, 1988; Robison, 2004). These jellyfish feed on a great variety of taxa, including euphausiids, cladocerans, and decapod zoea, fish (including fish larvae), and especially other jellyfish such as *Aurelia* spp. and ctenophores (Larson, 1986; Strand & Hamner, 1988; Towanda & Thuesen, 2006; Suchman et al., 2008). As prey, *Phacellophora* is consumed by a few animals like the giant deep-sea octopus *Haliphron atlanticus* (Hoving & Haddock, 2017) and sea anemones (P. Lovejoy, 2000, personal communication, in Ates, 2017). A panoply of organisms may also surround the bodies of the fried egg jellies, such as amphipods, juvenile crabs and fishes, and barnacles (Utinomi, 1968; Wrobel & Mills, 1998; Widmer, 2006; Suchman et al., 2008). Curiously, the sting of *Phacellophora* seems mild to humans (Mianzan, 1986).

We investigate the genetic diversity and phylogeographic history of *Phacellophora* species, with the available 16S and COI nucleotide sequences of samples from the NE Pacific, NW Atlantic, and the Azores. Through DNA barcoding, we corroborate and provide the first published report of *Phacellophora* occurring in the Azores. We also provide evidence of cryptic species within *Phacellophora* and discuss these results regarding morphological characteristics and the history of synonymy within the genus. We provide several suggestions for taxonomic rearrangements, from family to species level. Genetic tree inferences were interpreted conjunctly with the world reports of *Phacellophora*, to understand the occurrences of fried egg jellies in the temperate and sub-tropical NE Atlantic in recent decades.

## Materials and Methods

### Sampling and morphologic analyses

*Phacellophora* samples from the Azores were collected by João Rocha (x1) and BIM (x2), while snorkeling in Terceira and São Miguel islands, respectively, in June, October, and November 2020. The specimen collected in Terceira Island was frozen after collection and degraded. A piece of the remaining frozen tissue (ca. 2 cm) was later preserved in ethanol 96% for genetic analyses. Morphological characteristics were recorded, analyzed, and photographed in the two specimens collected in São Miguel Island while alive (by BIM), and later (by CJM and BIM) while preserved in formalin (ca. 4%) diluted in seawater. A small piece of the oral arm (ca. 2 cm) or gonad was isolated for genetic analyses. The *Phacellophora* collected in São Miguel were deposited in the biological collection of the University of the Azores-Campus of Horta (Faial Island). Sampling data of the *Phacellophora* subjected to genetic analyses are listed in Table 1.

**Table 1:** List of DNA sequences of *Phacellophora* used in this study, showing the genetic marker, accession number (from Genbank, or BOLD SYSTEMS if marked with an asterisk (*)), sampling location, latitude, longitude, sampling date, and bibliographic reference.

Marker	Accession number	Sampling location	Latitude	Longitude	Sampling date	Reference
COI	GQ120099	Gulf of Maine, New England, USA	42.29	−67.49	12 Sep 2007	Ortman et al. (2010)
COI	GQ120098	Gulf of Maine, New England, USA	42.29	−67.49	12 Sep 2007	Ortman et al. (2010)
COI	GQ120097	Gulf of Maine, New England, USA	42.29	−67.49	12 Sep 2007	Ortman et al. (2010)
COI	MF742371	Lincoln City, Oregon, USA	45	−124.55	Jul 2013	Abboud, Gómez-Daglio & Dawson (2018)
COI	MF742370	Lincoln City, Oregon, USA	45	−124.55	Jul 2013	Abboud, Gómez-Daglio & Dawson (2018)
COI	MF742369	Lincoln City, Oregon, USA	45	−124.55	Jul 2013	Abboud, Gómez-Daglio & Dawson (2018)
COI	MF742368	Lincoln City, Oregon, USA	45	−124.55	Jul 2013	Abboud, Gómez-Daglio & Dawson (2018)
COI	MF742367	Lincoln City, Oregon, USA	45	−124.55	Jul 2013	Abboud, Gómez-Daglio & Dawson (2018)
COI	MF742366	Lincoln City, Oregon, USA	45	−124.55	Jul 2013	Abboud, Gómez-Daglio & Dawson (2018)
COI	MF742365	Pillar Point, San Mateo County, California, USA	37.5	−122.75	22 Jul 2013	Abboud, Gómez-Daglio & Dawson (2018)
COI	MF742364	Pillar Point, San Mateo County, California, USA	37.5	−122.75	22 Jul 2013	Abboud, Gómez-Daglio & Dawson (2018)
COI	MF742363	Pillar Point, San Mateo County, California, USA	37.5	−122.75	22 Jul 2013	Abboud, Gómez-Daglio & Dawson (2018)
COI	MF742362	Pillar Point, San Mateo County, California, USA	37.5	−122.75	22 Jul 2013	Abboud, Gómez-Daglio & Dawson (2018)
COI	MF742361	Pillar Point, San Mateo County, California, USA	37.5	−122.75	22 Jul 2013	Abboud, Gómez-Daglio & Dawson (2018)
COI	MF742360	Pillar Point, San Mateo County, California, USA	37.5	−122.75	22 Jul 2013	Abboud, Gómez-Daglio & Dawson (2018)
COI	MF742359	Bell Harbor Marina, Washington, USA	47.61	−122.34	07 Jul 2012	Abboud, Gómez-Daglio & Dawson (2018)
COI	MF742358	Bell Harbor Marina, Washington, USA	47.61	−122.34	07 Jul 2012	Abboud, Gómez-Daglio & Dawson (2018)
COI	MF742357	Bell Harbor Marina, Washington, USA	47.61	−122.34	07 Jul 2012	Abboud, Gómez-Daglio & Dawson (2018)
COI	MF742356	Bell Harbor Marina, Washington, USA	47.61	−122.34	07 Jul 2012	Abboud, Gómez-Daglio & Dawson (2018)
COI	MF742355	Bell Harbor Marina, Washington, USA	47.61	−122.34	07 Jul 2012	Abboud, Gómez-Daglio & Dawson (2018)
COI	MF742354	Bell Harbor Marina, Washington, USA	47.61	−122.34	07 Jul 2012	Abboud, Gómez-Daglio & Dawson (2018)
COI	MF742353	San Quintín, Baja California, Mexico	30.34	−115.97	27 Aug 2009	Abboud, Gómez-Daglio & Dawson (2018)
COI	MF742352	San Quintín, Baja California, Mexico	30.34	−115.97	27 Aug 2009	Abboud, Gómez-Daglio & Dawson (2018)
COI	MF742351	San Quintín, Baja California, Mexico	30.34	−115.97	27 Aug 2009	Abboud, Gómez-Daglio & Dawson (2018)
COI	MF742350	San Quintín, Baja California, Mexico	30.34	−115.97	27 Aug 2009	Abboud, Gómez-Daglio & Dawson (2018)
COI	MF742349	San Quintín, Baja California, Mexico	30.34	−115.97	27 Aug 2009	Abboud, Gómez-Daglio & Dawson (2018)
COI	MF742348	San Quintín, Baja California, Mexico	30.34	−115.97	27 Aug 2009	Abboud, Gómez-Daglio & Dawson (2018)
COI	*KBCSM011-14	Vancouver Island, Canada	48.54	−123.54	28 May 2013	Hotke (2015)
COI	*KBCSM013-14	Vancouver Island, Canada	48.54	−123.54	28 May 2013	Hotke (2015)
COI	*KBCSM229-14	Hecate Strait, British Columbia, Canada	52.72	−129.8	30 May 2013	Hotke (2015)
COI	*KBCSM237-14	Hecate Strait, British Columbia, Canada	52.7	−130.05	30 May 2013	Hotke (2015)
COI	*KBCSM258-14	Hecate Strait, British Columbia, Canada	52.82	−130.78	31 May 2013	Hotke (2015)
COI	*KBCSM701-14	Vancouver Island, Canada	48.54	−123.54	28 May 2013	Hotke (2015)
COI	*KHBC182-13	Vancouver Aquarium, Canada	NA	NA	NA	Hotke (2015)
COI	*KBCSM472-14	Hecate Strait, British Columbia, Canada	53.96	−131.142	08 Jun 2013	Hotke (2015)
COI	*KBCSM473-14	Hecate Strait, British Columbia, Canada	53.96	−131.142	08 Jun 2013	Hotke (2015)
COI	MZ945512	Lagoa, São Miguel Island, Azores, Portugal	37.741225	−25.573640	19 Jun 2020	**Present study**
COI	MZ945513	Lagoa, São Miguel Island, Azores, Portugal	37.741226	−25.573641	30 Nov 2020	**Present study**
COI	MZ945514	Porto Judeu, Terceira Island, Azores, Portugal	38.643933	−27.131678	04 Oct 2020	**Present study**
16S	KY610658	Golfo de Panamá, Panama	8.98	−79.49	Jan 2012	Gómez-Daglio & Dawson (2017)
16S	JX393264	Bamfield, Canada	48.828125	−125.137511	21 April 2010	Sparmann, Ortman, Leander (2012, Direct submission, https://www.ncbi.nlm.nih.gov/nuccore/JX393264)
16S	JX393263	Bamfield, Canada	48.828125	−125.137511	12 Jun 2010	Sparmann, Ortman, Leander (2012, Direct submission, https://www.ncbi.nlm.nih.gov/nuccore/JX393263)
16S	JX393262	NE USA	42.297667	−67.498333	22 Sep 2003	Sparmann, Ortman, Leander (2012, Direct submission, https://www.ncbi.nlm.nih.gov/nuccore/JX393262)
16S	JX393261	Vancouver Aquarium, Canada	NA	NA	NA	Sparmann, Ortman, Leander (2012, Direct submission, https://www.ncbi.nlm.nih.gov/nuccore/JX393261)
16S	MZ947235	Lagoa, São Miguel Island, Azores, Portugal	37.741225	−25.573640	19 Jun 2020	**Present study**
16S	MZ947236	Lagoa, São Miguel Island, Azores, Portugal	37.741226	−25.573641	30 Nov 2020	**Present study**
16S	MZ947237	Porto Judeu, Terceira Island, Azores, Portugal	38.643933	−27.131678	04 Oct 2020	**Present study**

### DNA analyses

The *Phacellophora* tissue samples isolated for genetic analyses were processed conjunctly with ca. 1,000 other gelatinous samples, in the molecular lab of the University of the Azores-Campus of Horta, using fast, cheap, and high throughput laboratory methods.

Genomic DNA was extracted with the “QuickExtract™ DNA Extraction Solution” following the manufacturer protocol, except the volume of the reagent that was cut to half, as well as the amount of jellyfish tissue (ca. 0.5 cm^3^). The primers SHA (ACGGAATGAACTCAAATCATGT) and SHB (TCGACTGTTTACCAAAAACATA) (Cunningham & Buss, 1993), and LCO1490

(GGTCAACAAATCATAAAGATATTGG) and HCO2198 (TAAACTTCAGGGTGACCAAAAAATCA) (Folmer et al., 1994) were used to amplify ca. 600 and ca. 658 base pairs of the mitochondrial genes 16S and COI, respectively. A pool of different primer-tag combinations (designed by Srivathsan et al., 2019) was synthesized in addition to these primers, to identify, through demultiplexing, the corresponding PCR products after DNA sequencing. For PCR amplification we mixed 0.25–1 µl of DNA template, 0.4 µl of each primer, 6.5 µl of “Supreme NZYTaq II 2x Green Master Mix” (Nzytech, Lisbon, Portugal) and 4.5–5.25 µl of H_2_O. The PCR conditions were: 95 °C for 5 min (one cycle), followed by 34 cycles consisting of 94 °C for 30 s, 46.5 °C for 40 s, and 72 °C for 45 s, and a final extension at 72 °C for 5 min. The success of PCR reactions was verified through runs in agarose gels of 2 µl of each PCR product. Different volumes of the PCR products were later mixed, according to PCR amplification success, the scientific relevance of the genetic material, and, eventually, the success of the previous DNA sequencing run. We sequenced the PCR products in two runs with a MinION sequencer (©Oxford Nanopore Technologies, Oxford, United Kingdom), using the Ligation Sequencing Kit SQK-LSK109 during both runs, and an R9.4.1 flow cell and R10 flow cell on the first and second runs, respectively. ONTBarcoder v. 0.1.9.1 (Srivathsan et al., 2021) was used, using default settings, for demultiplexing and to obtain consensus sequences of DNA Barcodes. These consensus sequences were then verified and/or corrected in Geneious Prime® 2021.1, through the alignment (with MAFFT v7.450, using default options) of the consensus sequences with few sequences of systematically close taxa (for reference), plus the corresponding raw reads demultiplexed by the “miniBarcoder” software (Srivathsan et al., 2019). Raw reads less aligned and noted to correspond to contaminations of other taxa (after standard nucleotide “BLAST” searches), were excluded to get more accurate consensus sequences of the demultipled reads alignments. These consensus sequences were then contrasted to the consensus sequences obtained with the “ONTBarcoder” software.

The 16S and COI sequences of the *Phacellophora* from the Azores were then aligned, separated by marker, with MAFFT v7.450 in Geneious Prime® 2021.1 using default settings, along with sequences of congeners available in public databases. Nucleotide sequences of *Cyanea* and *Aurelia* were used as outgroups, respectively for the 16S and COI datasets. The resulting alignments were trimmed to the shortest sequence at each alignment end of the alignment.

The nucleotide alignments were submitted to PHYML V3.0 (Guindon et al., 2010; webserver: http://www.atgc-montpellier.fr/phyml/), for ML phylogenetic tree search, choosing the “Automatic model selection by SMS” (Lefort, Longueville & Gascuel, 2017), 1,000 standard bootstrap analyses, and available optimization options. For the COI tree search with PHYML, we added as input tree a Neighbor-Joining majority rule consensus tree, run for 1,000 bootstraps, and generated with GENEIOUS V2021.1. The resulting trees were finally edited in ITOL V5.0 (Letunic & Bork, 2021) and INKSCAPE V1.0.2.

Kimura 2-Parameter pairwise sequence distances (K2P PSD) were determined, for the COI alignment, with MEGA V7.026 (Kumar, Stecher & Tamura, 2016). Genetic distances were plotted in R V1.3 (R Core Team, 2020). A PSD species delimitation line of 6% was added to the plot graphic, as Abboud, Gómez-Daglio & Dawson (2018) suggested that value as a threshold to differentiate between inter-and intra- species COI sequence divergence for macromedusae.

Haplotypes of *Phacellophora* were determined with the DNA Collapser from FABOX V1.5 (Villesen, 2007) (Table S2) and confirmed with the “haplotypes” package from R V1.3 (R Core Team, 2020). The haplotypes were used to create a Median-Joining Network in POPART V1.7 *(*Leigh & Bryant, 2015) and mapped in R V1.3 (R Core Team, 2020) with geographical data of *P. camtschatica* sequences.

### Reports of *Phacellophora* observations

Geolocation data on *Phacellophora* observations and/or collection was obtained from various sources, namely: (1) scientific publications; (2) databases of the websites GBIF.org (2021); inaturalist.org, and jellywatch.org; (3) publications with *Phacellophora* photos reported in Facebook and Instagram; and (4) through direct correspondence with observers (see sightings details in Table S1). *Phacellophora* reports in social networks were inspected and validated. *Phacellophora* reports derived from scientific publications, museum specimens, or scientific expeditions, were later differentiated from the remaining observations, to control for the reliability of the data. The coordinates (sometimes extrapolated) of sampling/observation locations are listed in Table S1 and were mapped using R V1.3 (R Core Team, 2020) according to the data source. The data were then analyzed according to the oceanic region and report date.

## Results

### Morphological analysis

The diagnostic characters of the genus *Phacellophora* (Mayer, 1910; Jarms & Morandini, 2019) were confirmed in the two macromedusae collected in São Miguel Island (Azores) subjected to molecular analyses, namely: 16 rhopalia alternating with 16 clusters of subumbrellar tentacles, central stomach round with radiating branched and unbranched canals, marginal ring present, gonads hanging bellow the subumbrellar wall, and no subgenital pits.

According to the morphological characters defined by Mayer (1910) to diagnose the four nominal species of *Phacellophora* currently synonymized, our two fried egg jellies collected in São Miguel (Azores) would be classified as *P. sicula* (see Table 2).

**Table 2:** Synopsis of the putative diagnostic characteristics to differentiate the four nominal species of *Phacellophora* currently synonymized (*sensu* Mayer, 1910), excluding specimens collected excessively far from type localities.

Name	*P. camtschatica*	*P. ambigua* ^1^	*P. ornata* ^1^	*P. sicula* ^2^	Azores specimen col. June 2020	Azores specimen col. Nov. 2020	*Phacellora* Fedele (1937b)
**Diameter of disk in cm**	50 to 60	15 to 20	25–45	15.5	13.8 (11,6 in formalin)	17,7 (13,7 in formalin)	45
**Shape and number of marginal lappets**	16 trilobate lappets in rhopalar radii. Seven small lappets in each of 16 semicircular, velar lobes.	(4 × 16) 64 lappets, all similar to each other, and evenly rounded.	(4 × 16) to (6 × 16) lappets all similar to each other and evenly rounded.^3^	32 narrow, rounded rhopalar lappets (or 16 bilobate). 16 simple velar lobes.	As in *P. sicula*	As in *P. sicula*	16 bilobate rhopalar lappets. 4–7 × 16 small lappets in velar lobes
**Shape of mouth-arms.**	Long, narrow, resembling those of *Aurelia*.	Wide, curtain-like, and resembling those of *Cyanea*.	As in *P. ambigua*.	As in *P. ambigua*.	As in *P. ambigua*.	As in *P. ambigua*.	As in *P. ambigua*.
**Number of radial-canals**	16 branched rhopalar canals, and 5 × 16 unbranched	16 branched, rhopalar, and 3 to 5 × 16 unbranched	16 branched rhopalar, and 2 to 5 × 16 unbranched	16 branched, rhopalar, and 3 to 5 × 16 unbranched	16 branched rhopalar, and 3 to 5 × 16 unbranched	16 branched rhopalar, and 2 to 5 × 16 unbranched	16 branched rhopalar, and 3 to 5 × 16 unbranched
**Number of tentacles in each cluster**	20 to 24	9	5 to 9	9 to 15	9 to 21	5 to 21	14 to 24
**Where found**	North Pacific from Siberia to California	Pacific coast of North America	NW Atlantic	Mediterranean	Azores	Azores	Mediterranean

**Notes:**

The respective morphologic characteristics for the two *Phacellophora* collected in the Azores, and the specimen of Fedele (1937b), are included for comparison.

1 Closely allied, probably identical (*sensu* Mayer, 1910).

2 Intermediate in character between *P. camtschatica* and *P. ornata* (*sensu* Mayer, 1910).

3 Fedele (1937b) mentions bilobate rhopalar lappets, and 2–4 lappets in each of the 16 velar lobes (see also Fig. 395 of Mayer, 1910).

**Figure 4:**
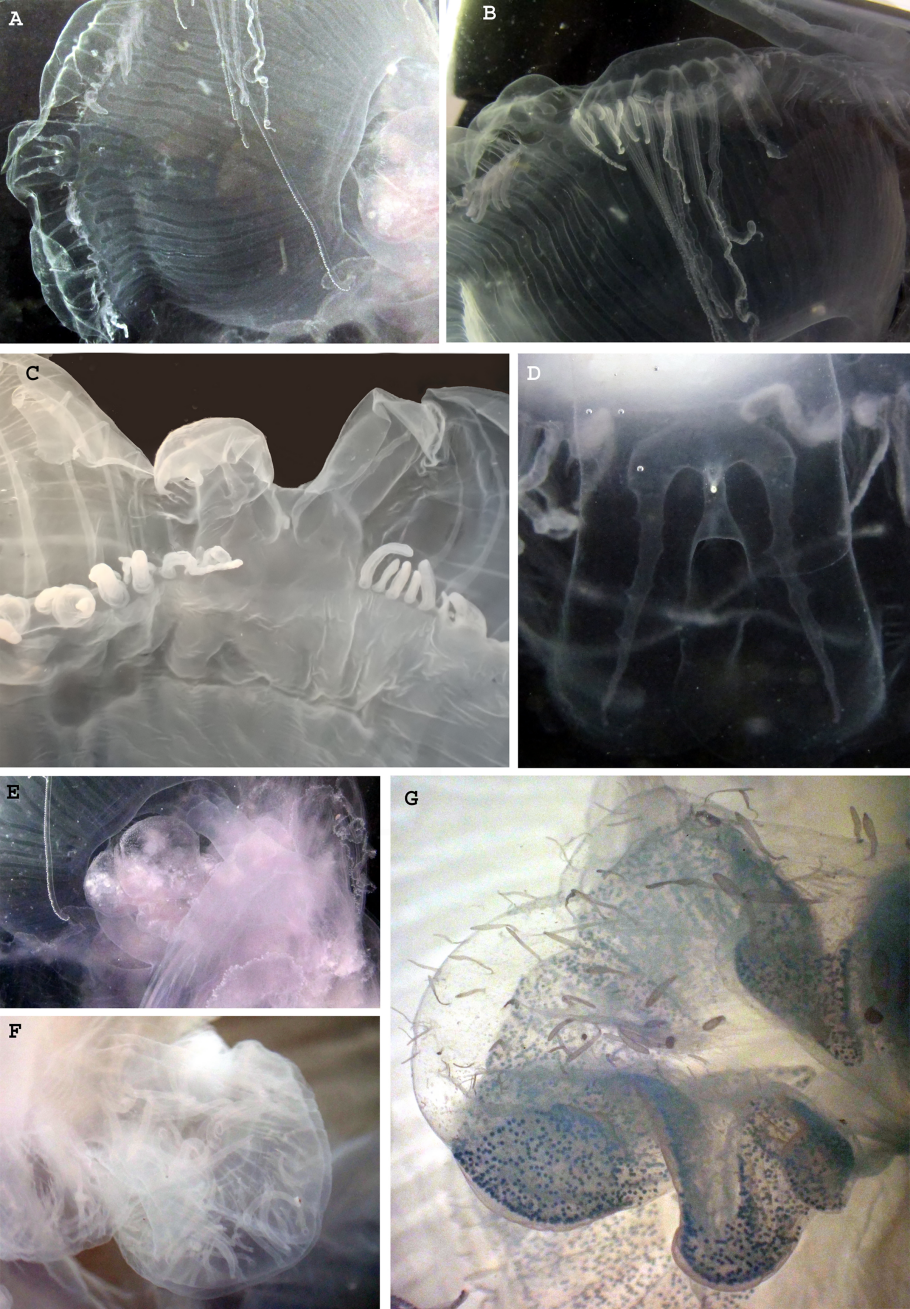
Morphological details of *Phacellophora* individuals collected in the Azores, and submitted to DNA barcode analyses. (A) Ropalium trifurcation (middle left), simple and branched radial canals extending in a centrifugal direction. (B) Typical cluster of tentacles. (C) Marginal lappets on rhopalium. (D) Rhopalium bellow marginal lappets. (E) Male gonad between oral arms. (F) Male gonad. (G) Female gonad. Credits: Bruno I. Magalhães (A, B, D, E), Carlos J. Moura (C, F, G).

Of the four nominal species of *Phacellophora* considered valid until Fedele (1937b), *P. camtschatica* (North Pacific) would present the most distinctive morphotype, for its long and narrow oral arms, and the three lappets per rhopalium (Table 2). *Phacellophora ambigua* (North Pacific) would be diagnosed by the four lappets per rhopalium, and wide curtain-like oral arms; the later character shared with Atlantic and Mediterranean congeners (Table 2). *Phacellophora ornata* (NW Atlantic) would be distinct for presenting all marginal lappets with similar size (or bilobate rhopalar lappets and 2–4 lappets per velar lobe, according to Fedele, 1937b), and *P. sicula* (type locality in the Mediterranean) would be the only species with one bilobate lappet per rhopalium and one lappet per velar lobe (Table 2). The Azores specimens presented consistently an arrangement of marginal lappets similar to that of *P. sicula* (Table 2). However, we further noted that the bilobate lappets of rhopalia, in the Azores specimens, fold but do not separate at one side from one of the adjacent velar lobes (Fig. 4C). Apparently, there is no correlation between the number of lappets and the diameter of the fried egg jellyfish (cf. Table 2). If the marginal lappets could diagnose *Phacellophora* species, the specimen collected by Fedele (1937b), with a distinctive arrangement of lappets of different sizes (Table 2), could potentially refer to a distinct species of *Phacellophora*. The characters “number of radial-canals” and “number of tentacles in each cluster” seem variable and identical between putative species (Table 2), thus inappropriate to diagnose these morphotypes.

**Figure 5:**
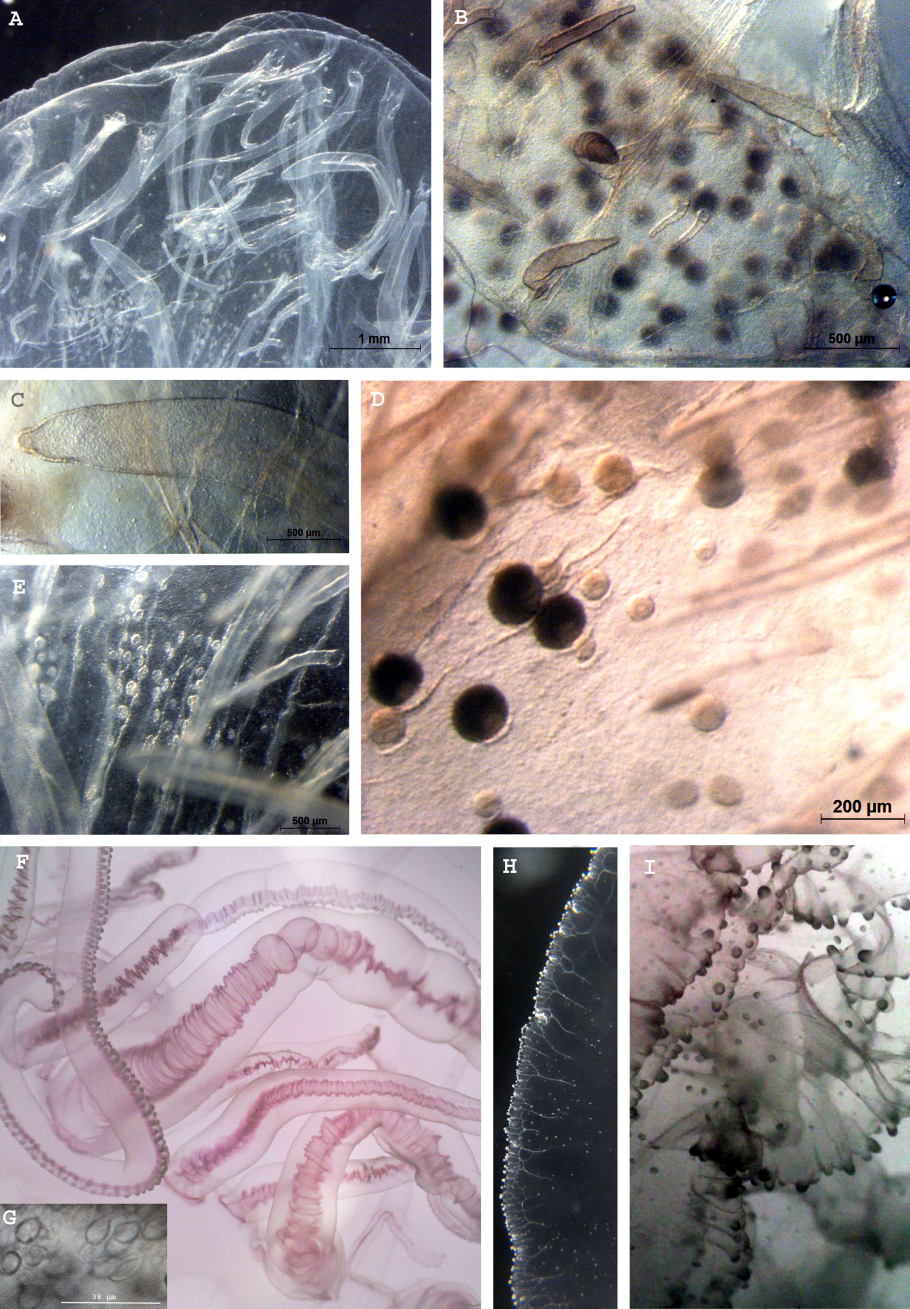
Morphological details of *Phacellophora* individuals collected in the Azores, and submitted to DNA barcode analyses. (A) Male gonad. (B) Female gonad. (C) Filament/cirri inside male gonad. (D) Female gametes in different stages. (E) Male gametes and cirri. (F) Tentacles. (G) Unidentified nematocysts present in tentacles and oral arms. (H, I) Margin of oral arm with digitate papillae filled with batteries of nematocysts. Photos credits: Carlos J. Moura (A–E), Bruno I. Magalhães (F–I).

The gonads of the male *Phacellophora* collected in June 2020 in São Miguel Island (Azores), presented sperm adherent to the inner walls of the conspicuous external pouches projected from the floor of the subumbrella and located between the four oral arms around the gastric opening (Figs. 4E, 4F). It is notable the presence of several cirri/filaments, apparently lacking nematocysts (Fig. 5C), inside the gonad pouches, protruding from the endodermal wall (Figs. 4E, 4F, 5A, 5C, 5E). In turn, the *Phacellophora* we collected in November 2020 revealed a female, with gonads (only inspected after formalin fixation) not much projected but more folded/evaginated, and containing oocytes in advanced and intermediate maturation stages that connect with the endoderm through a pedicel (Figs. 4G, 5B, 5D). Curiously, the cirri/filaments of this female species, apparently also lacking nematocysts, in this case, are disposed externally on the outer side of the ectoderm that protects the gonads (Figs. 4G, 5B, 5D).

### DNA analyses

**Figure 6:**
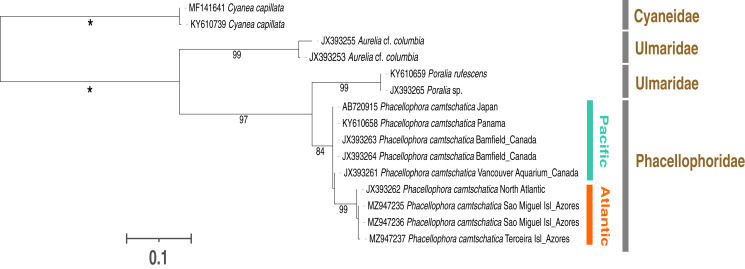
Maximum-likelihood phylogenetic tree (16S marker) of the *Phacellophora* genus (with the sequences of Ulmaridae and Cyaneidae as outgroup). Only bootstrap values between 70 and 100 percent are present. ‘*’: bootstrap values of 100%.

**Figure 7:**
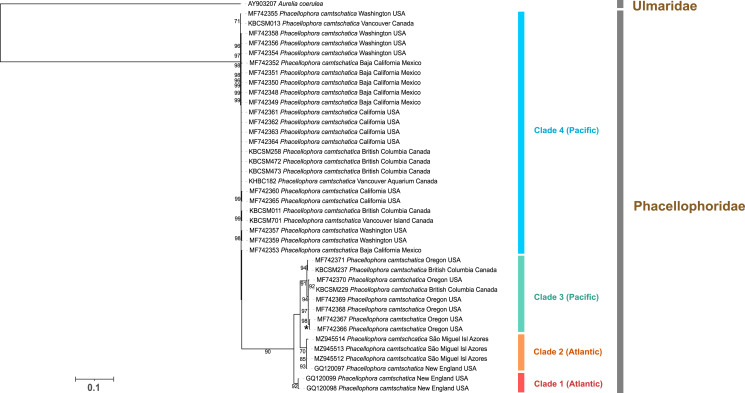
Maximum-likelihood phylogenetic tree (COI marker) of the *Phacellophora* genus (with a sequences of Ulmaridae as outgroup). Only bootstrap values between 70 and 100 percent are present. ‘*’: bootstrap values of 100%.

The sequencing of the 16S and COI mitochondrial markers corroborated the assignment of the three peculiar macromedusae collected in the Azores to the genus *Phacellophora* (Figs. 6, 7).

Phylogenetic analyses with 16S sequence data (Fig. 6) highlighted two distinctive sister clades of *Phacellophora* (3.3–4% K2P sequence divergence): one present in the NE and E Pacific (Canada and Panama), the other occurring in the NW Atlantic and the Azores. Curiously, two close 16S haplotypes found in the Azores diverge only 0.2–0.5% (K2P genetic distance) from the haplotype present in the NE of the USA. Additionally, the genus *Poralia* (Ulmaridae) was recovered as the sister group of the *Phacellophora* (Phacellophoridae), forming a clade in turn sister to the genus *Aurelia* (Ulmaridae). The family Ulmaridae is thus recovered paraphyletic.

**Figure 8:**
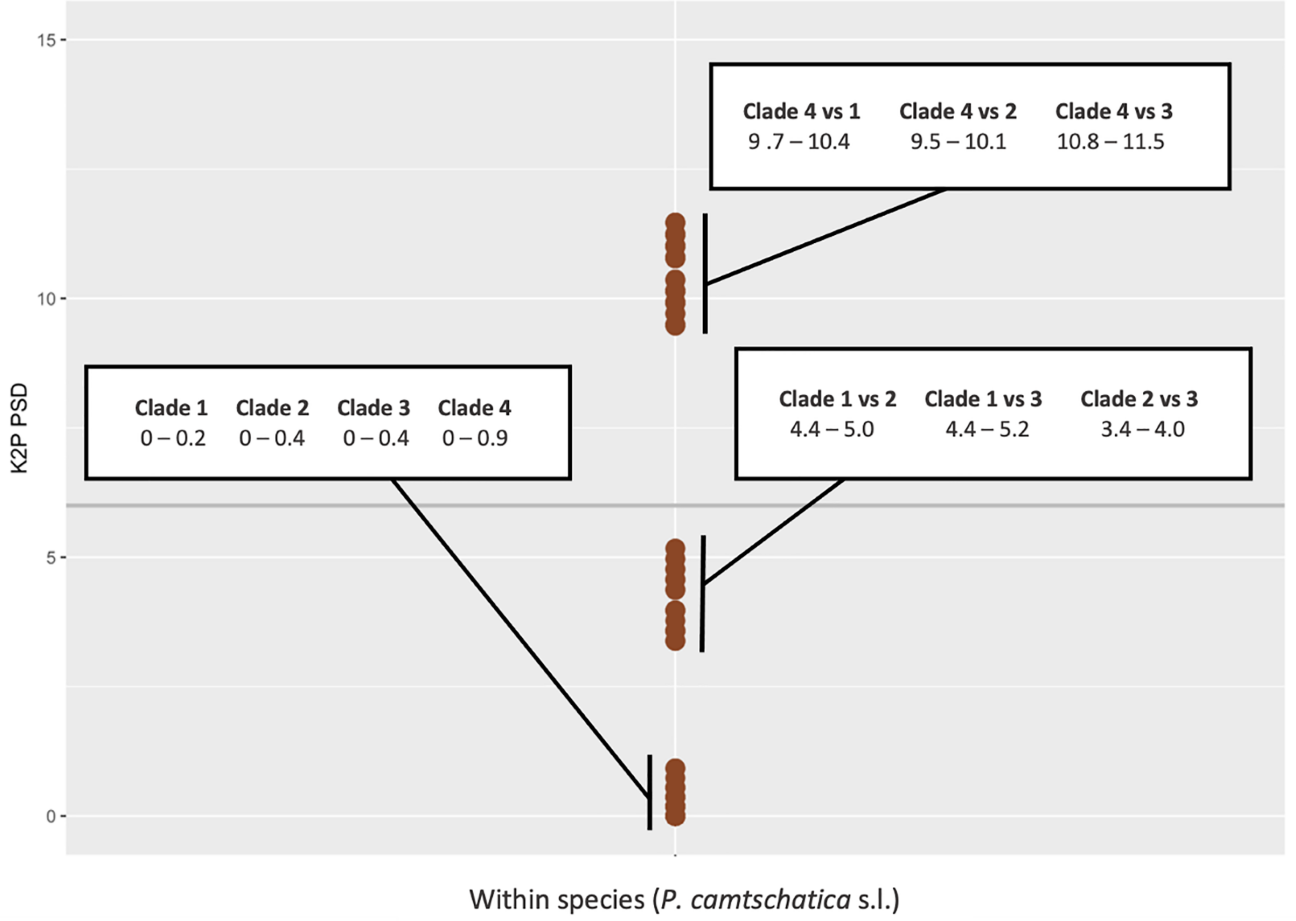
Kimura 2-Parameter (K2P) pairwise sequence divergences (PSD) for cytochrome c oxidase subunit I (COI) plotted between sequences of *Phacellophora camtschatica* s.l. Horizontal grey line: PSD = 6% (the threshold for species delimitation as suggested for macromedusae by Abboud, Gómez Daglio & Dawson, 2018).

COI sequence data, in turn, evidenced four main clades within the monophyletic *Phacellophora* genus (Fig. 7). Similarly to the 16S results, COI sequences of *Phacellophora* from the Azores and Gulf of Maine (NW Atlantic) group together with high similarity, and even share a COI haplotype (0–0.3% K2P distance; Fig. 7). Nevertheless, we identify another distinct Atlantic clade with two representatives from the NE of the USA. A third clade, exclusively represented by NE Pacific samples, groups with high support with the two Atlantic clades previously mentioned. A fourth clade of *Phacellophora* represented by 25 NE Pacific samples, presents the highest genetic divergence to the three other clades (Figs. 7, 8).

Assuming a threshold of 6% COI pairwise sequence divergence to delimit species of macromedusae when using the Kimura 2-Parameter (K2P), as suggested by Abboud, Gómez-Daglio & Dawson (2018), we may verify the genetic distances between the *Phacellophora* “clade 4” and the three other clades of *Phacellophora* represented (9.5–11.5% K2P), are clearly in the range of inter-species genetic distances of Ulmaridae (Figs. 7, 8). The genetic distances between clades 1, 2, and 3 are slightly below the threshold of 6% K2P (Fig. 8).

**Figure 9:**
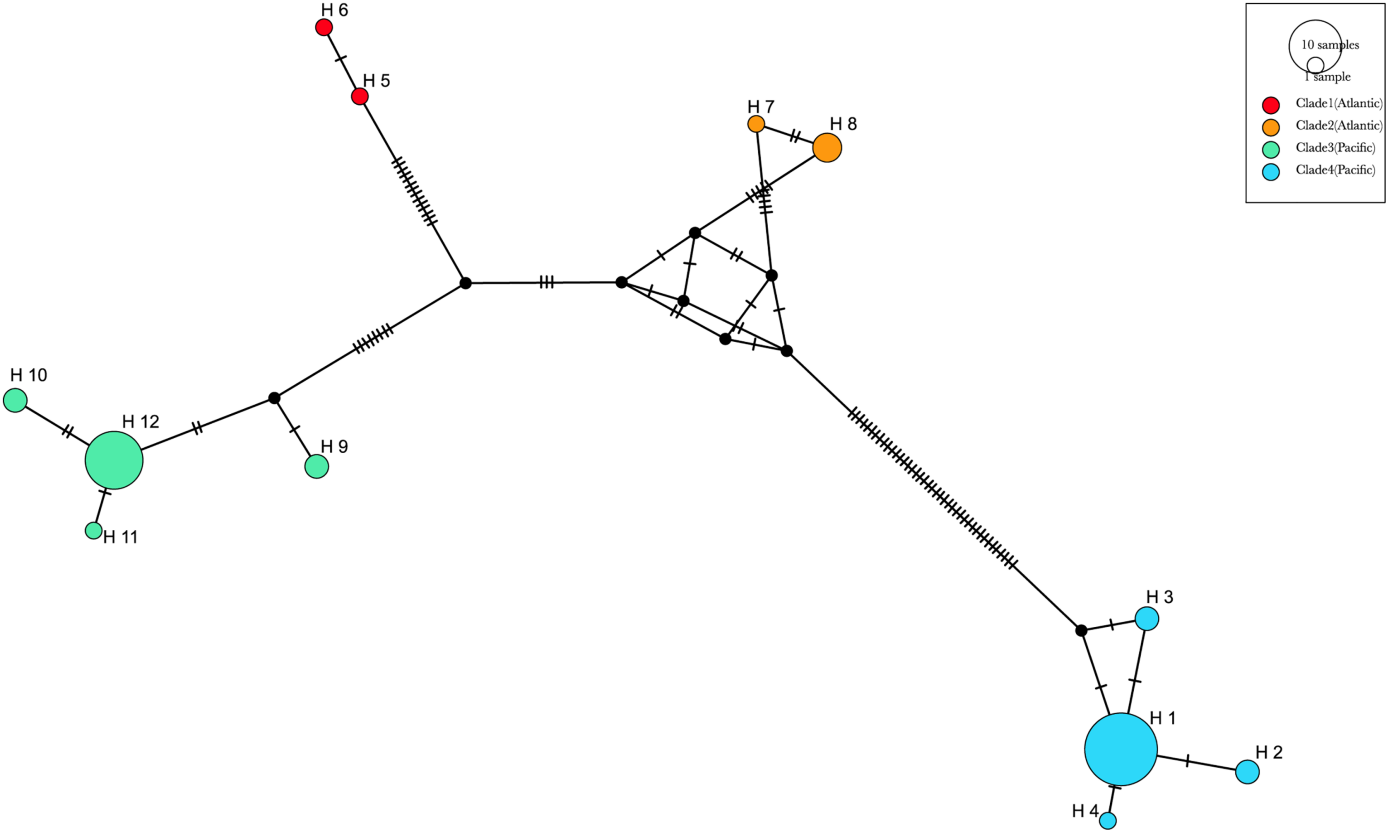
Haplotype median-joining network showing the phylogenetic relationships between COI haplotypes of the four main clades of *Phacellophora camtschatica* s.l. identified. Circle sizes are proportional to haplotype frequencies. The four main clades are identified by different colors (as assigned in Fig. 7). Transversal lines between haplotypes represent mutational steps.

The haplotype network analyses (Fig. 9) generated with the available 12 haplotypes of 39 COI sequences of *Phacellophora*, revealed similar patterns as those observed through the analyses of Fig. 7. The genetic distance between the Pacific “clade 4” and the three other clades is the highest, with 61 mutational steps from the Atlantic “clade 1”, 56 mutational steps from the Pacific “clade 3”, and 47 mutation steps from the Atlantic “clade 2”. The higher relatedness between the two Atlantic clades is not obvious with this analysis, but the distance between these two clades is smaller than with any of the other two Pacific clades. A similar haplotype network analysis without the divergent “clade 4” (results not shown), suggested a common ancestor for the Atlantic lineages.

**Figure 10:**
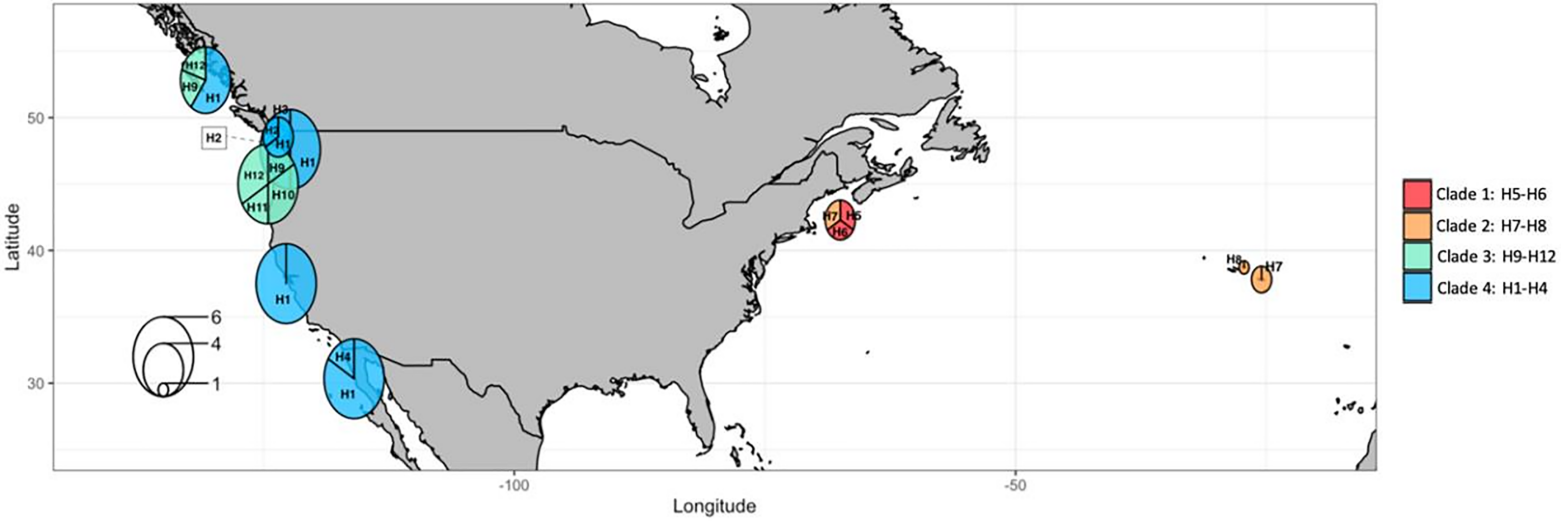
COI haplotype frequency map of clades of *Phacellophora camtschatica* s.l. (as designated in Fig. 7). Circle sizes are proportional to haplotype frequencies and colors refer to clades. Haplotype codes are shown for each main geographical area (check also Tables S2, S3).

The most frequent haplotype H1 (frequency of 51.28%; see Tables S2, S3) belongs to “clade 4”. The haplotype H1 is found in all the main Pacific regions represented (*i.e.*, British Columbia, Washington, California, and Baja California). In British Columbia (Canada) it co-exists with haplotypes of “clade 3” (H9 and H12) (Tables S2, S3). Curiously, in Oregon (NW, USA) only haplotypes of “clade 3” are represented (H9, 10, 11, and 12), but a little further north in Washington state (NW, USA) three haplotypes of “clade 4” are present (H1, H2, and H3). In Baja California, while H1 (“clade 4”) seems the predominant haplotype, it is the only place where H4 (of “clade 4”) was found. In the Gulf of Maine (NW Atlantic, USA), were found two haplotypes of “clade 1” (H5 and H6) and one haplotype of “clade 2” (H7) also present in the Azores. In the Azores, another haplotype of “clade 2” was found (H8). (see Figs. 9, 10).

## Discussion

### Morphological analyses

The two *Phacellophora* from the Azores inspected, which present high genetic affinity with a haplotype from the NW Atlantic, would be classified as *P. sicula* (type locality in the Mediterranean), if following the putative diagnostic characters established by Mayer (1910) to differentiate nominal species of *Phacellophora* currently synonymized. We observed in our specimens consistency in the number of lappets placed on and between rhopalar radii. Following the analysis of Fedele (1937b), the character of the disposition of marginal lappets, as well as the shape of mouth-arms, could be the most relevant characteristics to set apart *Phacellophora* morphotypes. Therefore, these characters should be further inspected in vouchers of the different lineages of *Phacellophora* DNA Barcoded, being potentially useful to diagnose and reassess the nominal species currently synonymized.

Additionally, we found peculiar the multiple filaments/cirri found, in the interior wall of the gonads of the male specimen, and on the external wall of the gonads of the female exemplar. These cirri are likely analogous to the “gastric filaments” identified inside the gastric chambers of several Semaeostomeae (*e.g.*, Russell, 1970), and to the four pairs of gastric filaments detected in ephyra of *Phacellophora* (Widmer, 2006). We did not detect nematocysts in these filaments, and thus we suspect these structures may act in the release and/or retention of gametes, as a potential reproductive strategy of these jellyfish.

### Taxonomy issues with *Phacellophora*

The present DNA barcoding analyses highlighted cryptic diversity within the nominal species *Phacellophora camtschatica*. There are at least two species of *Phacellophora* occurring in sympatry in the NE Pacific (a result already superficially noted in the MSc thesis of Hotke, 2015), with around 11% of genetic distance for COI (Fig. 8). Two other genetic lineages occurring in sympatry in the NW Atlantic may represent one or two additional distinct *Phacellophora* species (4.4–5% sequence divergence between the Atlantic clades, with the COI marker), considering the apparent segregation of genetic lineages between the N Pacific and NW Atlantic (Figs. 7–10) and the known reports of *Phacellophora* occurrences (Figs. 2, 3). However, few (somewhat dubious) reports of different *Phacellophora* morphotypes (*i.e., P. camtschatica* and *P. ornata*) in the Arctic (Fewkes, 1888) suggest sporadic sympatry of divergent lineages in that ocean, and the genetic distances of 3.4–5.2% between clades 1 to 3 fall below the threshold of 6% (suggested by Abboud, Gómez-Daglio & Dawson, 2018) to differentiate between intra- and inter- sequence divergence of macromedusae. Nevertheless, it is worth mentioning that Lawley et al. (2021) recently named and validated cryptic species of *Aurelia* diverging with smaller interspecific genetic distances (2–3.1% for COI), namely *A*. *cebimarensis* (from Brazil) and *A. smithsoniana* (from the Caribbean).

The apparent segregation of N Pacific and N Atlantic lineages suggests historical gene-flow disruption(s) through the Arctic, and likely the divergence of *Phacellophora* species between the Pacific and Atlantic. This would imply the revalidation of *Phacellophora ornata* (type locality in the NW Atlantic), and potentially also of *P. sicula* (type locality in the Mediterranean) if morphological diagnostic differences are consistent between morphotypes and respective genetic lineages. If it would not be the case, *P. ornata*, described firstly, would have nomenclatural priority over *P. sicula*.

The two *Phacellophora* collected in São Miguel Island (Azores), which revealed high genetic affinity with one NW Atlantic haplotype of “clade 2” (Fig. 7), presents higher morphologic affinity to the *P. sicula* morphotype originally described from the Mediterranean. Unfortunately, there is no COI or 16S sequences of *Phacellophora* from the Mediterranean, but the phylogeographic association between the Azores and the Mediterranean is not surprising in jellyfish (Ale et al., 2019; C. J. Moura et al., 2021, unpublished data). In the case of *Phacellophora*, populations of the NE Atlantic and the Mediterranean seem evolutionarily derived from the NW Atlantic, based on the published records of the fried egg jelly worldwide (cf. Figs. 2, 3) and phylogeographic evidence with current COI sequence data (Figs. 7, 9, 10). Nevertheless, only DNA sequences from the Mediterranean and the E Atlantic may validate that hypothesis. If in the future the *P. sicula* morphotype from the Mediterranean reveals close genetic association with “clade 2” (Azores and NW Atlantic), and the *P. ornata* morphotype eventually corresponds to “clade 1”, both nominal species could simultaneously be resurrected. Furthermore, the morphological dissimilarity of the *P. camtschatica* morphotype as compared to the three other morphotypes/species (*sensu* Mayer, 1910), suggests the genetically divergent “clade 4” could correspond to the *P. camtschatica* morphotype. It would also make sense that *P. ambigua* may correspond to “clade 3” and *P. ornata* to “clade 1” considering the morphologic similarity noted between these two morphotypes. Although, again, these hypotheses need validation through the morphologic inspection of multiple vouchers of the four main genetic lineages of *Phacellophora* herein identified. Nevertheless, our analyses provide clear evidence for at least two species of *Phacellophora*, and therefore at least *P. ornata*, originally described from the NW Atlantic and the second nominal species with taxonomic priority, should be resurrected, after investigating the interspecific relationships among morphologic and genetic characters.

Additionally, we call further attention to the invalidity of the family Phacellophoridae, an issue already questioned by Gómez-Daglio & Dawson (2017) and evidenced in phylogenetic hypotheses of Bayha et al. (2010), Abboud, Gómez-Daglio & Dawson (2018), and this study (Fig. 6). According to the phylogenetic positions and genetic distances between genera of the order Semaeostomeae, the genera *Phacellophora* and *Poralia* may instead be united in a subfamily of the Ulmaridae. This subfamily could be the Sthenoniinae Mayer, 1910 (as suggested by Mayer, 1910; Kramp, 1961; Larson, 1986; and more recently Gómez-Daglio & Dawson, 2017), but only if molecular phylogenetic analyses confirm a close phylogenetic relationship between the genera *Phacellophora*, *Poralia*, and *Sthenonia*. Currently, the monotypic genera *Poralia* and *Sthenonia* are placed alone in the subfamilies Poraliinae Larson, 1986 and Sthenoniinae, respectively (WoRMS Editorial Board, 2021). According to Mayer (1910), the subfamily Sthenoniinae would include the three genera, due to the synapomorphic trait of the disposition of the tentacles in linear clusters from the floor of the subumbrella. These genera also present four unbranched mouth-arms, and evaginated, sac-like gonads without subgenital pits (Mayer, 1910). However, while *Poralia* and *Phacellophora* are closely related morphologically (Mayer, 1910) and genetically (Gómez-Daglio & Dawson, 2017), the genus *Sthenonia* presents eight rhopalia (Mayer, 1910) and thus may be more distantly related. Only molecular analyses including *Sthenonia* will clarify if the three genera should belong to a single subfamily (*i.e.*, Sthenoniinae), to two subfamilies (*i.e.*, Sthenoniinae and Poraliinae, with *Phacellophora* in the latter), or even three subfamilies (*i.e.*, Sthenoniinae, Poraliinae, and Phacelophoriinae) to denote the marked morphologic peculiarities of the three genera.

As a final note, we stress that the identification of some uncommon jellyfish (and many other cnidarians and invertebrates) based solely on visual/photo observations should be viewed with great skepticism. Doubt about the accuracy of visual/photo identifications should be particularly high when the species in question has not been the subject to DNA barcoding and/or detailed laboratory morphological analyses of specimens. Ideally, both morphological and genetic characters should be examined, considering the pervasion of cryptic diversity in marine invertebrates. For example, in this study, upon first observations of *Phacellophora* in the Azores, different jellyfish experts gave different opinions about the species identification based solely on photographs. After the recognition of *Phacellophora* in the Azores through morphological analyses and DNA barcoding, the posterior confirmation of the taxon based on photos is more reliable. The jellyfish species known in the NE Atlantic or the Mediterranean that are at least somewhat similar are *Drymonema dalmatinum, D. gorgo*, and *Cyanea capillata*. While the latter presents a bell margin divided into eight pairs of thick lobes (*e.g.*, Cowles & Cowles, 2007), *Drymonema* species may be easily distinguished by the “bell markings of central circle with red/brownish bifurcating radiating lines” and an unclustered arrangement of tentacles (cf. Öztürk, 2020 and Ciriaco, Faresi & Segarich, 2021, and contrast with Fig. 1). We suspect that some reports of *D. dalmatinum* in the Mediterranean may refer to *Phacellophora* which seems much more widespread in that basin than is assumed. Similarly, some confusion might have occurred in identifications, especially for the Cape Verde area, between *Phacellophora* and *Drymonema gorgo* (originally described from Brazil). Likewise, some registers of *Phacellophora* provided in Table S1 and Figs. 2, 3, based solely on photographs, may also potentially correspond to other morphologically similar species.

An obvious solution to address the high uncertainty associated with many jellyfish reports worldwide would be the training and hiring of more jellyfish experts to accurately catalog and monitor jellyfish presence and dynamics.

### Phacellophora reports worldwide

The fried egg jellyfish *Phacellophora* has been more often reported in the North Pacific, where it has always been considered common (*e.g.*, Brotz, 2011; Il’inskii & Zavolokin, 2011; Uye & Brodeur, 2017), but apparently has become more frequent in some areas (*e.g.*, Siddon & Zador, 2017; Uye & Brodeur, 2017; plus some comments in social networks of citizens inhabiting NE Pacific shores). Unpublished observations of *Phacellophora* may exist for the western Indian Ocean, and perhaps even the Central Pacific Ocean (compared to Fig. 2), as illustrated in the distribution map of *P. camtschatica* by Jarms & Morandini (2019). The most striking confirmed reports are those from recent years in the Mediterranean (Mills et al., 1996; Dragičević et al., 2019; present study) and especially in the NE Atlantic during the past two to three decades (Moro et al., 2020; Wirtz, 2021; present study; Fig. 3; Table S1).

Sightings of *Phacellophora* in the Azores apparently only started in 2012 (three observations in different islands), then in 2013 (one obs.), 2015 (three obs. in two islands), 2020 (around 20 obs. in four islands), and so far we only know of three in 2021 (one island). All these sightings are from divers/snorkelers in shallow waters (except one exemplar found stranded on a beach), during all seasons. Of note, aggregations of *Phacellophora* were observed in Terceira Island, in July and September 2020, and in June 2021. In 2021 the presence and abundance of gelatinous fauna in the Azores, including the “mauve stinger” *Pelagia* jellyfish, was considerably less than that of 2020, which could potentially relate to the dramatic decrease of *Phacellophora* reports in the Azores along 2021.

According to Moro et al. (2020), *Phacellophora* was observed in all islands of the Canaries on over 20 occasions, after 1994, year-round. The first available sighting of *Phacellophora* in Madeira dates to 2006 (M. Kaufmann, 2006, personal communication; Table S1). Reports in the Gibraltar area are relatively recent (1991, 2014, 2018, 2019, and 2020), like those from the west coast of mainland Portugal (in 2020 and 2021). Interestingly, we found a photograph of *Phacellophora* taken in northern Spain in 2007 (see Table S1).

**Figure 11:**
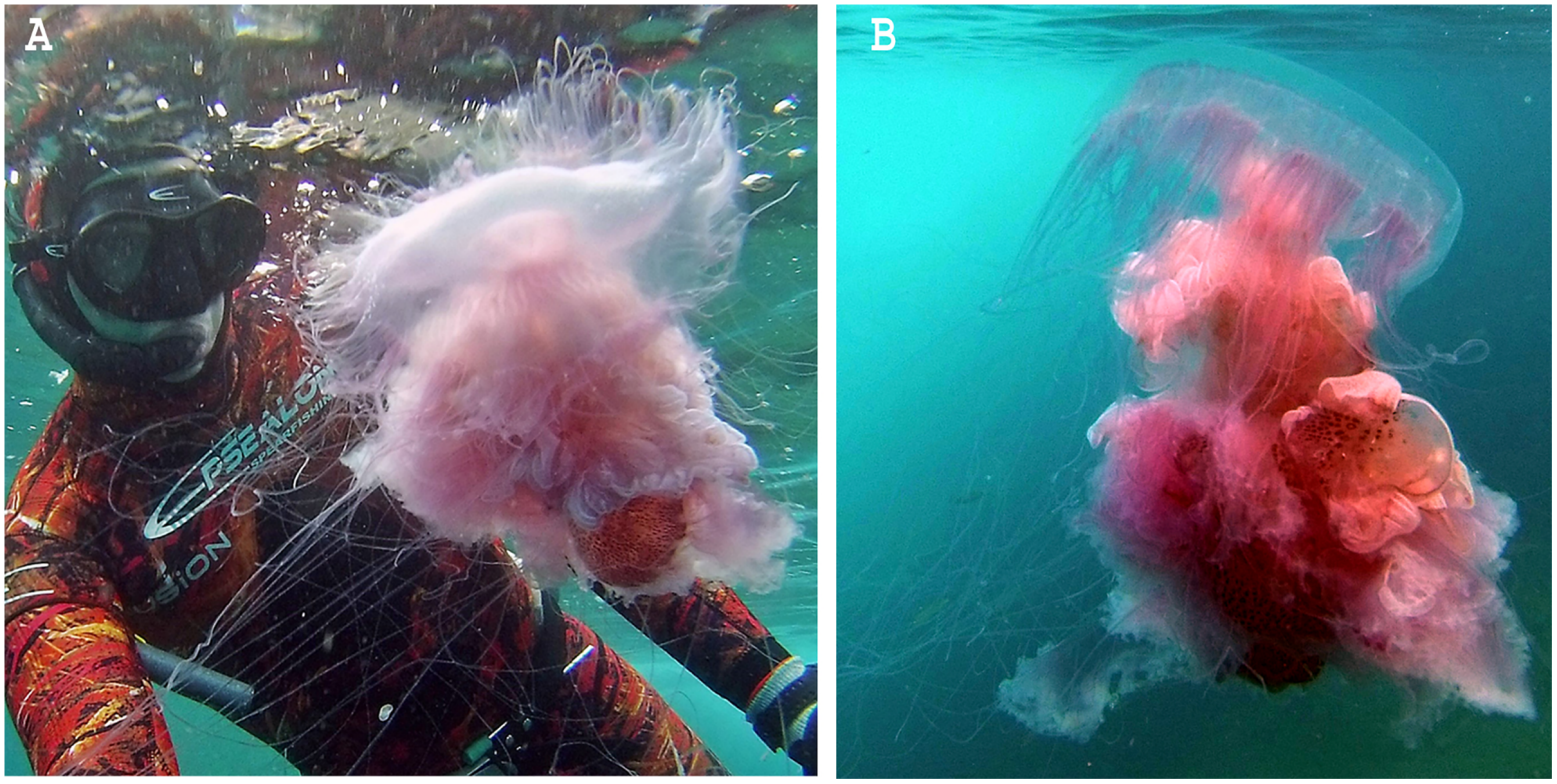
*Phacellophora* sp. predating on *Pelagia noctiluca* at the Azores (NE Atlantic). Photos credits: João P. Rocha.

*Phacellophora* preys on moon jellies *Aurelia* spp. and ctenophores in the Pacific (Strand & Hamner, 1988), but now we present evidence of *Phacellophora* preying on *Pelagia noctiluca* in the Azores (J. P. Rocha, 2020, personal communication; Fig. 11). Therefore, the apparent increase in frequency and duration of *P. noctiluca* blooms in the Mediterranean and NE Atlantic (Daly et al., 2010; Licandro et al., 2010; Canepa et al., 2014) could correlate with the recent outbreak of *Phacellophora* in that oceanic region. This might be a strong possibility, as *Aurelia* are not common in Macaronesia, and we show that despite very rare observations of *Phacellophora* in the Mediterranean and NE Atlantic around the early-mid 20^th^ century, this conspicuous taxon was recorded much more often in the past two decades (cf. Table S1 and Figs. 2, 3). Analogously, the sudden occurrences of the medusivorous *Drymonema* in the Mediterranean also seemed to be correlated with outbreaks of its prey: the “moon jelly” *Aurelia* (Malej et al., 2014). Nevertheless, we do not discard other possible reasons for these recent outbreaks of *Phacellophora*, such as oceanic currents, water temperature, or nutrients.

It is noteworthy that the close genetic relatedness of *Phacellophora* from the cold NW Atlantic waters and the sub-tropical Azores seas (Figs. 6, 7), suggests not only the adaptability of a supposedly cold-water jellyfish lineage to warm-mild sea temperatures but also relatively recent genetic connectivity between distant areas where the taxon was not frequently observed. This could be explained by a hypothetical colonization of the NE Atlantic (and possibly the Mediterranean) from the NW Atlantic, considering the apparent absence (or eventual weak presence) of *Phacellophora* in the Indian Ocean, but also the three reports of *Phacellophora* in high seas at west and northwest of the Azores, in 1910 (Kramp, 1948) and 1978 (www.gbif.org) respectively.

The apparent recent proliferation of the fried egg jellyfish, in the past two to three decades, especially in the Macaronesia, the Iberian Peninsula, and the Mediterranean waters is of concern. While the medusivorous habits of *Phacellophora* may be beneficial to help to control the increasingly large populations of *Pelagia noctiluca* that severely impact fisheries and tourism in these areas, the recent expansion of the macromedusae *Phacellophora* in the NE Atlantic and the Mediterranean may also threaten the natural spatial-temporal dynamics of zooplankton, fishes and the local biodiversity. *Phacellophora* was already reported to negatively affect farming facilities of salmon in Chile (Palma, Apablaza & Soto, 2007) and fisheries (Bosch-Belmar et al., 2021), meaning the outbreaks of fried egg jellyfish represent a threat to aquaculture and fishing industries.

## Conclusions

The present study confirms and reports the occurrence of *Phacellophora* in the Azores, during the past two past decades, in coincidence with recent reports of the genus in other areas of the temperate to sub-tropical waters of the NE Atlantic, where it had not been observed before.

The predation of *Phacellophora* on the mauve stinger *Pelagia noctiluca*, confirmed in this study, could explain the recent increased number of *Phacellophora* sightings across the Macaronesia and the Mediterranean (but this hypothesis needs to be tested), as *P. noctiluca* seems to become increasingly more frequent in these oceanic regions. While the fried egg jellyfish populations could control the plague of *P. noctiluca*, and its stings may be harmless to humans, an increasing number of *Phacellophora* in the NE Atlantic and the Mediterranean also threaten fisheries, aquaculture, and the local biodiversity.

The presence of *Phacellophora* in the Azores, and likely in the NE Atlantic and the Mediterranean, may derive from a recent migration (in terms of geological time) from the cold waters of the NW Atlantic, as suggested through occurrence reports of the taxon and by the great similarity between 16S and COI haplotypes from the NW Atlantic and the Azores.

We highlighted cryptic diversity within the nominal species *Phacellophora camtschatica*, namely three to four cryptic species within the monophyletic genus *Phacellophora*. These results suggest the likely need for resurrection of nominal species currently synonymized, the designation of neotypes, and/or even the description of new taxa. Further haplotype sampling, and comprehensive morphological analyses of vouchers, are needed to verify the reliability of putative diagnostic characters, and posteriorly to proceed to taxonomic rearrangements. The number and arrangement of the marginal lappets of the umbrella may differentiate the four main *Phacellophora* morphotypes recognized by Mayer (1910), and thus this character should be inspected in specimens submitted to DNA barcoding.

Additionally, the genus *Phacellophora* needs to be moved to the family Ulmaridae. However, genetic data for *Sthenonia* are needed in order to decide in which subfamily *Phacellophora* should be placed.

The 16S marker was an excellent complement to COI, to DNA barcode jellyfish, due to its ease of PCR amplification with standard primers, and utility to investigate phylogenetic associations not resolved solely with the COI.

Finally, ideally, worldwide monitoring, cataloging, and DNA barcoding of macromedusae, should increase to enlighten the phylogeographic history and the extent of cryptic diversity of jellyfish and proceed to necessary taxonomic improvement. This work should be integrative and is only achievable through the training and hiring of jellyfish experts.

## Supplemental Information

10.7717/peerj.13125/supp-1 Supplemental Information 1 Reports of *Phacellophora* worldwide. (Note that some unavailable data could not be included, *e.g.*, Zavolokin, Glebov & Kosenok, 2008; Il’inskii & Zavolokin, 2011)

10.7717/peerj.13125/supp-2 Supplemental Information 2 Haplotype frequencies of *Phacellophora camtschatica* s.l.

10.7717/peerj.13125/supp-3 Supplemental Information 3 List of Haplotypes of *P. camtschatica* s.l. (colors match the COI clades).

## Additional Information and Declarations

### Data Availability

The following information was supplied regarding data availability: The six partial sequences of mitochondrial COI and 16S genes are available in the Supplemental Files and at GenBank: MZ945512–MZ945514 and MZ947235–MZ947237.
