# Supplementary material for: Insight into the cryptic diversity and phylogeography of the peculiar fried egg jellyfish Phacellophora (Cnidaria, Scyphozoa, Ulmaridae)
Source: PeerJ. 2022 Mar 31;10:e13125. doi: 10.7717/peerj.13125 (PMC8977069; doi:10.7717/peerj.13125)
Supplement: Supplemental Information 2 [file peerj-10-13125-s002.pdf]

| Accession number | Localisation | Longitude | Latitude | Haplotype |
|---|---|---|---|---|
| **GQ120099** | Gulf of Maine, New England, USA | -67.49 | 42.29 | H5 |
| **GQ120098** | Gulf of Maine, New England, USA | -67.49 | 42.29 | H6 |
| **GQ120097** | Gulf of Maine, New England, USA | -67.49 | 42.29 | H8 |
| **MF742371** | Lincoln City, Oregon, USA | -124.55 | 45 | H9 |
| **MF742370** | Lincoln City, Oregon, USA | -124.55 | 45 | H11 |
| **MF742369** | Lincoln City, Oregon, USA | -124.55 | 45 | H12 |
| **MF742368** | Lincoln City, Oregon, USA | -124.55 | 45 | H12 |
| **MF742367** | Lincoln City, Oregon, USA | -124.55 | 45 | H10 |
| **MF742366** | Lincoln City, Oregon, USA | -124.55 | 45 | H10 |
| **MF742365** | Pillar Point, San Mateo County, California, USA | -122.75 | 37.5 | H1 |
| **MF742364** | Pillar Point, San Mateo County, California, USA | -122.75 | 37.5 | H1 |
| **MF742363** | Pillar Point, San Mateo County, California, USA | -122.75 | 37.5 | H1 |
| **MF742362** | Pillar Point, San Mateo County, California, USA | -122.75 | 37.5 | H1 |
| **MF742361** | Pillar Point, San Mateo County, California, USA | -122.75 | 37.5 | H1 |
| **MF742360** | Pillar Point, San Mateo County, California, USA | -122.75 | 37.5 | H1 |
| **MF742359** | Bell Harbor Marina, Puget Sound, Washington, USA | -122.34 | 47.61 | H2 |
| **MF742358** | Bell Harbor Marina, Puget Sound, Washington, USA | -122.34 | 47.61 | H1 |
| **MF742357** | Bell Harbor Marina, Puget Sound, Washington, USA | -122.34 | 47.61 | H2 |
| **MF742356** | Bell Harbor Marina, Puget Sound, Washington, USA | -122.34 | 47.61 | H1 |
| **MF742355** | Bell Harbor Marina, Puget Sound, Washington, USA | -122.34 | 47.61 | H3 |
| **MF742354** | Bell Harbor Marina, Puget Sound, Washington, USA | -122.34 | 47.61 | H1 |
| **MF742353** | San Quint'n, Baja California Sur, Mexico | -115.97 | 30.34 | H4 |
| **MF742352** | San Quint'n, Baja California Sur, Mexico | -115.97 | 30.34 | H1 |
| **MF742351** | San Quint'n, Baja California Sur, Mexico | -115.97 | 30.34 | H1 |
| **MF742350** | San Quint'n, Baja California Sur, Mexico | -115.97 | 30.34 | H1 |
| **MF742349** | San Quint'n, Baja California Sur, Mexico | -115.97 | 30.34 | H1 |
| **MF742348** | San Quint'n, Baja California Sur, Mexico | -115.97 | 30.34 | H1 |
| ***KBCSM011-14** | Vancouver Island, Canada | -123.54 | 48.54 | H1 |
| ***KBCSM013-14** | Vancouver Island, Canada | -123.54 | 48.54 | H3 |
| ***KBCSM229-14** | Hecate Strait, British Columbia | -129.8 | 52.72 | H12 |
| ***KBCSM237-14** | Hecate Strait, British Columbia | -130.05 | 52.7 | H9 |
| ***KBCSM258-14** | Hecate Strait, British Columbia | -130.78 | 52.82 | H1 |
| ***KBCSM701-14** | Vancouver Island, Canada | -123.54 | 48.54 | H1 |
| ***KBCSM472-14** | Hecate Strait, British Columbia | -131.142 | 53.96 | H1 |
| ***KBCSM473-14** | Hecate Strait, British Columbia | -131.142 | 53.96 | H1 |
| **PCA1** | Sao Miguel Island, Azores | -25.4730853 | 37.8085274 | H8 |
| **PCA3** | Sao Miguel Island, Azores | -25.4730853 | 37.8085274 | H8 |
| **PCA7** | Terceira island, Azores | -27.2177696228027 | 38.7212104797363 | H7 |
| ***KHBC182-13** | Vancouver Aquarium, Canada | | | H1 |
