# Supplementary material for: Insight into the cryptic diversity and phylogeography of the peculiar fried egg jellyfish Phacellophora (Cnidaria, Scyphozoa, Ulmaridae)
Source: PeerJ. 2022 Mar 31;10:e13125. doi: 10.7717/peerj.13125 (PMC8977069; doi:10.7717/peerj.13125)
Supplement: Supplemental Information 3 [file peerj-10-13125-s003.pdf]

Haplotype	Frequency (in %)
H1	51.28
H2	5.13
H3	5.13
H4	2.56
H5	2.56
H6	2.56
H7	2.56
H8	7.7
H9	5.13
H10	5.13
H11	2.56
H12	7.7
Total	100
